# Bridging the Gap in End Tuberculosis Targets in the Elderly Population in Eastern China: Observational Study From 2015 to 2020

**DOI:** 10.2196/39142

**Published:** 2022-07-29

**Authors:** Kui Liu, Zhenhua Xie, Bo Xie, Songhua Chen, Yu Zhang, Wei Wang, Qian Wu, Gaofeng Cai, Bin Chen

**Affiliations:** 1 Zhejiang Provincial Center for Disease Control and Prevention Hangzhou China; 2 Ningbo Yinzhou No 2 Hospital Ningbo China; 3 School of Urban Design Wuhan University Wuhan China

**Keywords:** pulmonary tuberculosis, elderly population, prediction

## Abstract

**Background:**

With a progressive increase in the aging process, the challenges posed by pulmonary tuberculosis (PTB) are also increasing for the elderly population.

**Objective:**

This study aimed to identify the epidemiological distribution of PTB among the elderly, forecast the achievement of the World Health Organization’s 2025 goal in this specific group, and predict further advancement of PTB in the eastern area of China.

**Methods:**

All notified active PTB cases aged ≥65 years from Zhejiang Province were screened and analyzed. The general epidemiological characteristics were depicted and presented using the ArcGIS software. Further prediction of PTB was performed using R and SPSS software programs.

**Results:**

Altogether 41,431 cases aged ≥65 years were identified by the surveillance system from 2015 to 2020. After excluding extrapulmonary TB cases, we identified 39,832 PTB cases, including laboratory-confirmed (23,664, 59.41%) and clinically diagnosed (16,168, 40.59%) PTB. The notified PTB incidence indicated an evident downward trend with a reduction of 30%; however, the incidence of bacteriologically positive cases was steady at approximately 60/100,000. Based on the geographical distribution, Quzhou and Jinhua Cities had a higher PTB incidence among the elderly. The delay in PTB diagnosis was identified, and a significantly prolonged treatment course was observed in the elderly. Moreover, a 50% reduction of PTB incidence by the middle of 2024 was predicted using a linear regression model. It was found that using the exponential smoothing model would be better to predict the PTB trend in the elderly than a seasonal autoregressive integrated moving average model.

**Conclusions:**

More comprehensive and effective interventions such as active PTB screening combined with physical checkup and succinct health education should be implemented and strengthened in the elderly. A more systematic assessment of the PTB epidemic trend in the elderly population should be considered to incorporate more predictive factors.

## Introduction

Tuberculosis (TB) ranks 13th among the leading causes of death globally and is also the top cause of death from a single infectious agent, which has been a substantial public health concern and an urgent global public health priority [1,2]. Nearly a quarter of the global population is infected with *Mycobacterium tuberculosis*, although only <10% of the people developed active TB during their lifetime, especially in low- and middle-income areas [3-5]. According to the Global Tuberculosis Report 2021 released by the World Health Organization (WHO), an estimated 9.9 million people developed illness with an incidence of 127/100,000 in 2020 [6]. Meanwhile, the latest report has demonstrated that the estimated number of newly diagnosed TB cases was 842,000 with a notified incidence of 59/100,000 in China, which still ranks second among the 30 highly burdened countries [6]. Despite being a preventable and curable disease, it has serious effects on some targeted groups such as the elderly and continues to be a major challenge.

With progressive population aging in China, the population aged >60 years had reached 264 million based on the 7th population census, among which 190 million were >65 years old, accounting for 13.5% of the total population [7]. Recently, evidence indicated a progressive increase in the notification rate of pulmonary TB (PTB) with age, particularly in East Asian and Southeast Asian countries [8]. The geriatric population is susceptible to the development of TB, which may be attributed to reactivation of the lesions and change in immunity levels [9]. With the implementation of the directly observed treatment strategy, the prevalence of PTB in China declined sharply, whereas the exploration of the change trend in the elders indicated a paucity of data [10,11]. Therefore, exploring the potential characteristics of PTB among the elderly is necessary.

Zhejiang Province, a developed area located in Eastern China, has an economic output of exceeding US $1.08 trillion. According to the Zhejiang Provincial Health Commission in 2019, the elderly population aged ≥60 years reached 11,217,200, accounting for 22.43% of the total population of the province, which was 4.53% higher than that at the national level.

This study aimed to identify the epidemiological distribution of PTB among the elderly, assess the performance of the WHO’s 2025 goal in this specific group, and predict the further advancement of PTB in Eastern China. It would contribute to filling the existing policy gaps, prompting optimization of the current health policy, and constructing a new strategic health framework to realize the end TB targets.

## Methods

### Overview

This study was focused on Zhejiang Province, which is in the eastern region of China and comprises the following cities: Hangzhou, Ningbo, Wenzhou, Jiaxing, Huzhou, Shaoxing, Jinhua, Quzhou, Zhoushan, Taizhou, and Lishui. Its location and demographic information have been presented in previous studies [12,13]. Additionally, according to the latest data released by the Zhejiang Bureau of Statistics, there were 65.4 million permanent residents, of which 9.26 million were aged ≥65 years and accounted for 14.2% of the provincial population. The demographic data of this specific population showed an increase of 0.9% over the previous year [14].

### Data Collection

All PTB cases recorded in the Zhejiang Province during 2015-2020 were selected and screened from the TB information management system (TBIMS). The TBIMS was designed and constructed as a first-generation web-based information system by the National Center for TB Control and Prevention in 2005 [15]. All users of the TBIMS including designated hospitals, communities, and the Center for Disease Control and Prevention at the provincial, city, and county levels were authorized to fulfill their duties in this specific system [16]. Patients with PTB aged ≥65 years were included, and the data pertaining to their demographics, diagnosis, laboratory outcomes, and treatment outcomes were collected. Additionally, the basic data of Zhejiang Province were approved and obtained from the Chinese Information System for Disease Control and Prevention and local statistical yearbook [12].

### Definition

In this study, the PTB cases consisted of laboratory-confirmed PTB (also called bacteriologically diagnosed PTB) and clinically diagnosed PTB. The former is diagnosed by bacteriological evidence acquired by sputum smear and culture or a rapid diagnostic system, such as the GeneXpert MTB/RIF. The latter is diagnosed by chest imaging, epidemiological findings, and clinical symptoms along with other relevant testing [17]. All identifications are based on the National Diagnostic Criteria for Pulmonary Tuberculosis (WS288-2008 and WS288-2017) and Classification of Tuberculosis (WS196-2001 and WS196-2017) in China [18,19].

### General Characteristics of PTB in the Elderly

According to bacteriological and clinical diagnoses, the results are presented as the following parameters: sex, age groups, different cities, occupations, source of patients, treatment classification, anti-TB treatment, drug sensitivity results, treatment outcomes, and various delays in seeking treatment. Additionally, the notified incidence and spatial distribution depict the epidemiological characteristics of PTB in the elderly.

### Predictive Model for PTB in the Elderly

Based on the WHO’s 2025 goal, a 50% reduction in TB morbidity and 75% decline in the absolute number of TB deaths should be achieved. PTB in Zhenjiang Province was predicted using 2 methods. In method I, the available notified incidence was used to fit and screen regression models according to the R^2^ value. The linear regression model (LRM), exponential curve model, and growth curve model (GCM) were considered. These models are described in our previous study [20]. In method II, the seasonal autoregressive integrated moving average (ARIMA) model and exponential smoothing (ETS) method, 2 commonly used time series models, were compared to determine the appropriate one for predicting the trend of PTB cases in the elderly. The seasonal ARIMA and ETS models were used not only for short-term prediction but also for middle- and long-term forecasting [21,22]. The former consisted of p, d, and q and seasonal parameters p, d, and q, where p represents autoregression, d represents the degree of difference, and q represents the order of the moving average [23]. Furthermore, considering the patterns of addition, multiplication, or no patterns, the ETS model consisted of three vital parameters: error, trend, and seasonal components [24]. The formulas of the seasonal ARIMA and ETS models have been presented elsewhere, so they were omitted here [24,25]. For each method, the optimal model was selected based on the indicators of the Akaike information criterion, the corrected Akaike information criterion, and Bayesian information criterion [26-28]. Ultimately, the root mean squared error (RMSE) and mean absolute percentage error (MAPE) were used to compare the accuracy of prediction [24].

### Ethics Approval

Information on all the cases was fully anonymized in the processing of data. This study was approved by the Ethics Committee of the Zhejiang Provincial Center for Disease Control and Prevention (2021-027-01). As only public health surveillance data were used, the requirement of informed consent was waived by the abovementioned ethics committee. Moreover, all details used in this research were in accordance with the Law of the Prevention and Treatment of Infectious Diseases in the People’s Republic of China.

### Statistical Analysis

Descriptive analysis was performed using the R software (version 3.5.3, R Foundation for Statistical Computing), and the map was developed using the ArcGIS software (version 10.2, Esri). Additionally, the prediction of the PTB epidemic in 2025 was performed using SPSS Statistics 20.0 (IBM Corp), and the time series calculations involving the ETS and seasonal ARIMA models were performed using the R software (version 3.5.3). Statistical significance was set at *P*<.05.

## Results

### Notified Incidence and Geographical Distribution

**Figure 1:**
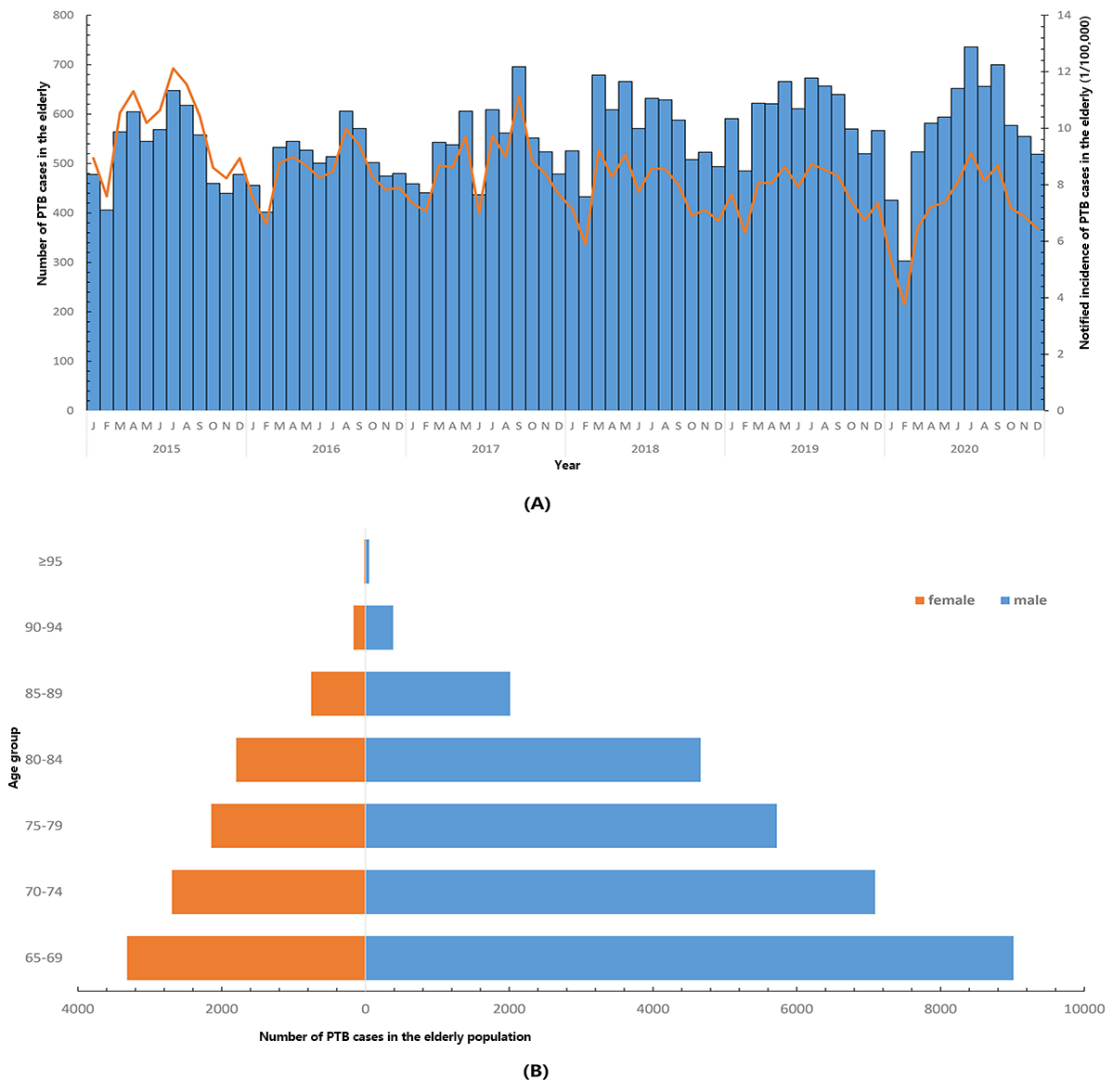
General epidemiological characteristics of PTB in the elderly: (A) Change trend of notified PTB incidence and recorded case number by months. (B) Distribution of notified PTB cases in different sexes by various age groups. PTB: pulmonary tuberculosis.

**Figure 2:**
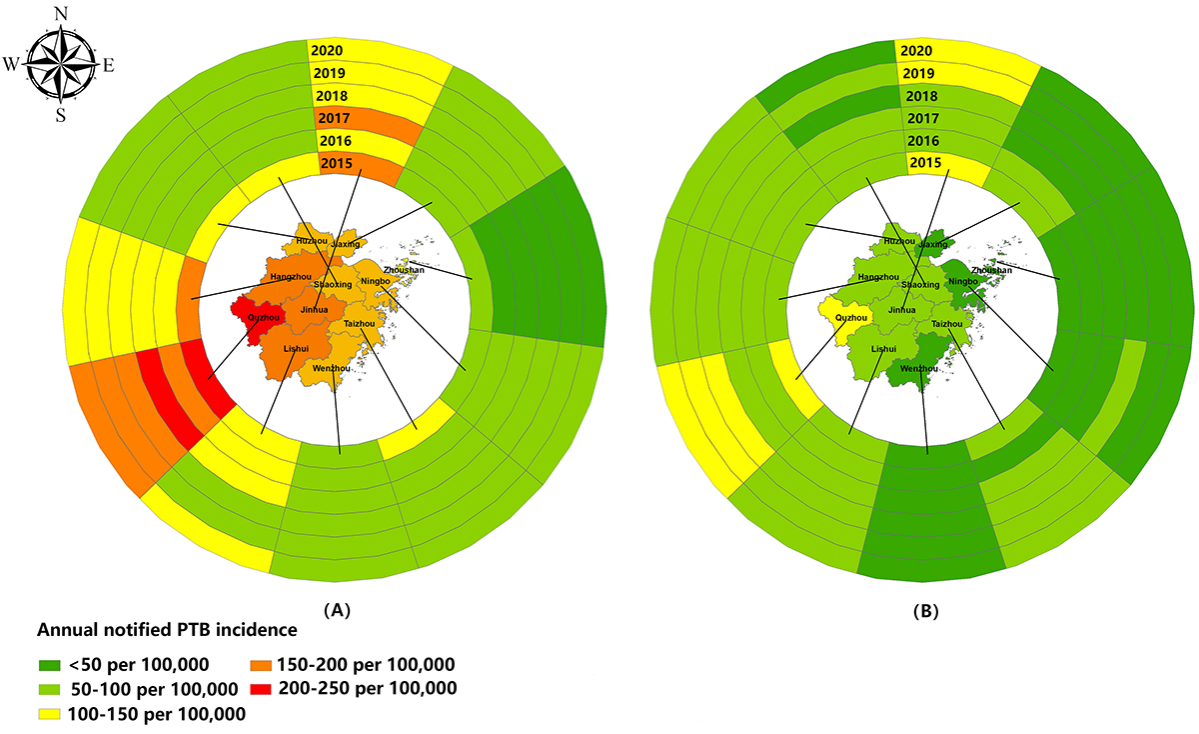
Geographical distribution of PTB cases in the elderly population during the study period: (A) Annual notified PTB incidence among different cities. (B) Annual bacteriologically positive PTB incidence among different cities. PTB: pulmonary tuberculosis.

A total of 41,431 records of people with PTB aged ≥65 years were notified in the TBIMS from 2015 to 2020. After excluding cases of extrapulmonary TB, we collected 39,832 PTB cases consisting of laboratory-confirmed PTB (23,664, 59.41%) and clinically diagnosed PTB (16,168, 40.59%), with the highest registered number in 2019 (n=7223). The notified incidence of PTB among the elderly was 119.09 per 100,000 in 2015; 100.66 per 100,000 in 2016; 103.44 per 100,000 in 2017; 93.26 per 100,000 in 2018; 93.74 per 100,000 in 2019; and 84.67 per 100,000 in 2020 (Figure 1A). The notified incidence of bacteriologically positive PTB cases among the elderly was 61.18 per 100,000 in 2015; 50.77 per 100,000 in 2016; 52.75 per 100,000 in 2017; 59.98 per 100,000 in 2018; 64.29 per 100,000 in 2019; and 58.98 per 100,000 in 2020. The former revealed an evidently declining trend, whereas the latter had a comparatively stable state. In terms of the geographical distribution of PTB in the elderly, Quzhou City had the highest incidence, whereas Zhoushan City had the lowest PTB burden. Moreover, the notified PTB incidence in the elderly illustrated an overall slow downward trend in various cities but still exceeded that in the general population (nearly 40 per 100,000 to 50 per 100,000) (Figure 2A). For bacteriologically positive cases, the notified incidence demonstrated a significant difference in various cities, where the incidence in Quzhou City was 5 times more than that in Zhoushan City (data not shown). Meanwhile, incidence of bacteriologically positive cases revealed significant differences among the 11 cities (Figure 2B). In addition, a U-trend was revealed for bacteriologically positive PTB incidence in Quzhou and Jinhua Cities, whereas a successive reduction was recorded in Jiaxing City. However, despite a relatively low notified incidence, Wenzhou City presented a steady upward trend. Additionally, nearly 9 cities demonstrated a unimodal trend for bacteriologically positive incidence in 2018-2019.

### General Characteristics of PTB in the Elderly

Of all the 39,832 geriatric PTB cases, men accounted for 72.67% (28,944 cases), whereas women accounted for 27.33% (10,888 cases). Regarding age distribution in each sex, the onset number in males was more than that in females, and a significant declining trend was observed, in which the age group of 65-69 years accounted for 30.96% (12,332) of the cases (Figure 1B). The median age was 73 years in both sexes. Regarding geographical distribution, Hangzhou, Jinhua, Taizhou, and Quzhou cities contributed to >10% among all notified PTB cases, in which the highest bacteriological (4514, 11.33%) and clinical diagnoses (3866, 9.71%) of PTB were both in the Hangzhou region. The top 3 occupations of the elderly were farming and working; being a retiree; and housekeeping, housework, and unemployment. Besides, referral and passive finding (also called actively seeking a doctor) had accounted for nearly 86% (34,284) patients among diverse sources. Most of all the elderly PTB cases (35,177, 88.31%) belonged to the initial treatment category, and 51.26% (20,418) comprised the bacteriological diagnoses. Nearly all the elderly patients could be provided anti-TB treatment, and 59.28% (23,613) received standardized treatment based on bacterial evidence. From the available information, the known drug resistance ratio was >2.7%, the drug resistance rate (rifampicin monoresistance) was 5.86% among the people aged ≥65 years, and the treatment success rate among the study population including completion of the treatment course and cure was approximately 72%. A longer delay was observed in the interval from the disease onset to the hospital visit compared to the interval from visiting the designated hospital to confirmation of PTB. Surprisingly, nearly half of the elderly cases had a treatment time >9 months (Table 1).

**Table 1:** Epidemiological characteristics of pulmonary tuberculosis cases among the elderly population in Eastern China from 2015 to 2020 (N=39,832).

Characteristic		Total PTB^a^	Laboratory-confirmed PTB	Clinically diagnosed PTB
**Sex, n (%)**				
	Male	28,944 (72.67)	17,347 (43.55)	11,597 (29.11)
	Female	10,888 (27.33)	6317 (15.86)	4571 (11.48)
**Age group (years), n (%)**				
	65-69	12,332 (30.96)	6670 (16.75)	5662 (14.21)
	70-74	9782 (24.56)	5809 (14.58)	3973 (9.97)
	75-79	7868 (19.75)	4826 (12.12)	3042 (7.64)
	80-84	6461(16.22)	4161 (10.45)	2300 (5.77)
	85-89	2769 (6.95)	1783 (4.48)	986 (2.48)
	90-94	551 (1.38)	380 (0.95)	171 (0.43)
	≥95	69 (0.17)	35 (0.09)	34 (0.09)
**City, n (%)**				
	Hangzhou	8380 (21.04)	4514 (11.33)	3866 (9.71)
	Ningbo	3797 (9.53)	2383 (5.98)	1414 (3.55)
	Wenzhou	3803 (9.55)	2120 (5.32)	1683 (4.23)
	Jiaxing	2343 (5.88)	1579 (3.96)	764 (1.92)
	Huzhou	2120 (5.32)	1331 (3.34)	789 (1.98)
	Shaoxing	3306 (8.30)	1998 (5.02)	1308 (3.28)
	Jinhua	5661 (14.21)	3676 (9.23)	1985 (4.98)
	Quzhou	3994 (10.03)	2256 (5.66)	1738 (4.36)
	Zhoushan	401 (1.01)	190 (0.48)	211 (0.53)
	Taizhou	4021 (10.09)	2400 (6.03)	1621 (4.07)
	Lishui	2006 (5.04)	1217 (3.06)	789 (1.98)
**Occupation, n (%)**				
	Farmer and worker	29,342 (73.66)	17,696 (44.43)	11,646 (29.24)
	Retiree	6128 (15.38)	3451 (8.66)	2677 (6.72)
	Housekeeping, housework, and unemployment	3406 (8.55)	1957 (4.91)	1449 (3.64)
	Unknown	512 (1.29)	330 (0.83)	182 (0.46)
	Others	284 (0.71)	151 (0.38)	133 (0.33)
	Commercial service stratum	97 (0.24)	51 (0.13)	46 (0.12)
	Cadre staff	37 (0.09)	16 (0.04)	21 (0.05)
	Herder	15 (0.04)	8 (0.02)	7 (0.02)
	Doctor	11 (0.03)	4 (0.01)	7 (0.02)
**Source of patients, n (%)**				
	Health examination	176 (0.44)	77 (0.19)	99 (0.25)
	Contact screening	3 (0.01)	2 (0.01)	1 (0)
	Passive finding	14,391 (36.13)	8174 (20.52)	6217 (15.61)
	Referral	19,893 (49.94)	11,889 (29.85)	8004 (20.09)
	Tracing	5205 (13.07)	3423 (8.59)	1782 (4.47)
	Others	164 (0.41)	99 (0.25)	65 (0.16)
**Classification of treatment, n (%)**				
	Initial treatment	35,177 (88.31)	20,418 (51.26)	14,759 (37.05)
	Retreatment	4655 (11.69)	3246 (8.15)	1409 (3.54)
**Anti-TB treatment, n (%)**				
	Yes	39,753 (99.8)	23,613 (59.28)	16,140 (40.52)
	No	74 (0.19)	51 (0.13)	23 (0.06)
**Results of drug sensitivity test, n (%)**				
	Monodrug resistance	866 (2.17)	817 (2.05)	49 (0.12)
	Polydrug resistance	3 (0.01)	2 (0.01)	1 (0)
	Sensitivity	17,289 (43.40)	16,422 (41.23)	867 (2.18)
	Multidrug resistance	208 (0.52)	195 (0.49)	13 (0.03)
	No test	21,466 (53.89)	6228 (15.64)	15,238 (38.26)
**Treatment outcome, n (%)**				
	Completion of the treatment course^b^	13,977 (35.09)	1546 (3.88)	12,431 (31.21)
	Cure^c^	14,380 (36.1)	14,335 (35.99)	45 (0.11)
	Death	2854 (7.17)	2171 (5.45)	683 (1.71)
	Failure	429 (1.08)	348 (0.87)	81 (0.20)
	Adverse reaction	598 (1.50)	316 (0.79)	282 (0.71)
	Transfer to MDR-TB^d^ treatment	254 (0.64)	230 (0.58)	24 (0.06)
	Others	7340 (18.43)	4718 (11.84)	2622 (6.58)
**Interval between onset and visit to the designated hospital (days), n (%)**				
	0-14	17,315 (43.47)	10,586 (26.58)	6729 (16.89)
	15-29	7944 (19.94)	4410 (11.07)	3534 (8.87)
	30-44	5417 (13.60)	3074 (7.72)	2343 (5.88)
	45-59	1826 (4.58)	992 (2.49)	834 (2.09)
	≥60	7284 (18.29)	4580 (11.50)	2704 (6.79)
	Unknown	46 (0.12)	22 (0.06)	24 (0.06)
**Interval between visit to designated hospital and confirmation of PTB (days), n (%)**				
	0-14	34,253 (85.99)	20,198 (50.71)	14,055 (35.29)
	15-29	2911 (7.31)	1746 (4.38)	1165 (2.92)
	30-44	1090 (2.74)	680 (1.71)	410 (1.03)
	45-59	458 (1.15)	280 (0.70)	178 (0.45)
	≥60	1053 (2.64)	718 (1.80)	335 (0.84)
	Unknown	67 (0.17)	42 (0.11)	25 (0.06)
**Interval between confirmation of PTB and end of therapy (days), n (%)**				
	<180	5059 (12.7)	3537 (8.88)	1522 (3.82)
	180-270	12,597 (31.63)	7644 (19.19)	4953 (12.43)
	≥270	17,224 (43.24)	9148 (22.97)	8076 (20.28)
	Unknown	4952 (12.43)	3335 (8.37)	1617 (4.06)

^a^PTB: pulmonary tuberculosis.

^b^Completion of the treatment course referred to the following situations: (1) Patients with negative etiology finished the standardized course with negative results of sputum smear/culture or no test was performed. (2) Patients with positive etiology finished the standardized course without undergoing a sputum result at the end of the treatment whereas the last sputum smear or culture yielded a negative result.

^c^Cure referred to the situation where patients with positive etiology finished the standardized course of treatment and had continuous negative results of sputum smear or culture in the last month and during the penultimate test.

^d^MDR-TB: multidrug-resistant TB.

### Trend Prediction for PTB in the Elderly

Based on the WHO’s 2025 goal, a reduction of 50% in TB incidence and 75% in TB absolute numbers should be achieved during the period from 2015 to 2025. All the 3 included models were considered (model with *P*<.05 for ANOVA). Considering a minor difference in R^2^, GCM and LRM (Table 2) were reserved for the prediction of notified PTB incidence. Eventually, the result indicated a possible achievement of the desired TB incidence in the middle of 2024 using LRM. Besides, given a considerable decline in PTB-related deaths in 2020, no model to predict the notified PTB death number based on available data was established.

**Table 2:** Predictive models for pulmonary tuberculosis incidence in the elderly.

Model	*F* (*df*1,*df2*)	*P* value	R^2^	Coefficients			
				b0	*P* value	b1	*P* value
LRM^a^	23.80 (1,4)	<.001	0.86	11619.97	<.001	–5.71	<.001
GCM^b^	27.69 (1,4)	<.001	0.87	119.27	<.001	–0.06	<.001

^a^LRM: linear regression model.

^b^GCM: growth curve model.

### Predictive Model for PTB Cases in the Elderly

**Figure 3:**
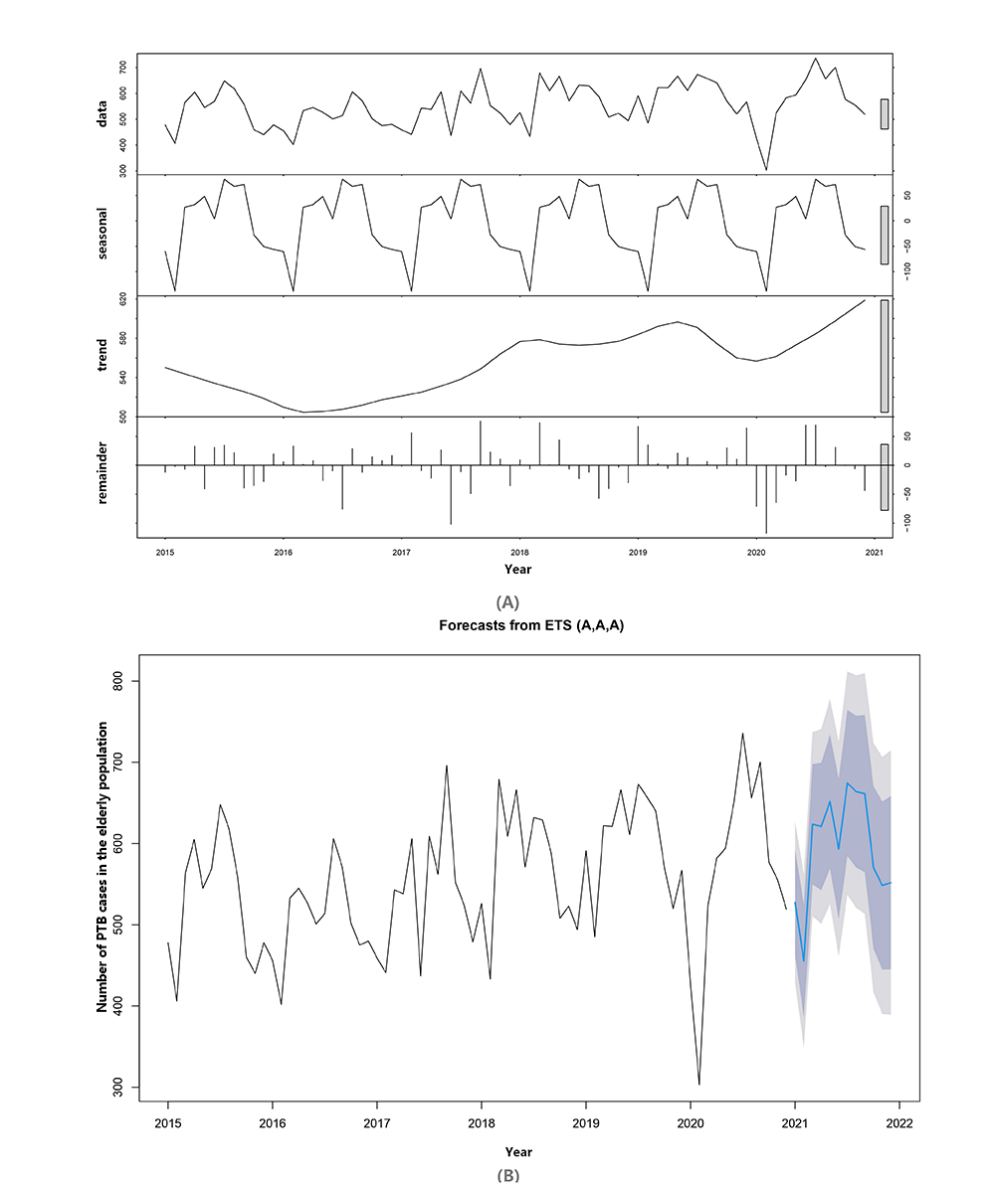
Composition of notified PTB cases in the elderly and prediction using the Holt-Winters exponential smoothing model. (A) Sequence composed of the seasonal effect, common trend, and random fluctuation (also called remainder). (B) Predicted number of the elderly PTB cases in 2021. ETS: exponential smoothing; PTB: pulmonary tuberculosis.

For comparing fitness to forecasting of the PTB epidemic trend among the elderly in 2021, the Holt-Winters exponential smoothing model (H-W ETS) demonstrated better predictive performance with lower RMSE and MAPE values than the seasonal ARIMA model (Table 3). The composition of the original sequence and the predictive results determined using the H-W ETS model are presented in Figure 3.

For this specific group, the authentic number of notified PTB cases was included in the 95% CI and nearly 91.7% included in the 80% CI, as observed in Table 4.

**Table 3:** Critical indices of the Holt-Winters exponential smoothing model and seasonal autoregressive integrated moving average model.

Model	RMSE^a^	MAPE^b^
H-W ETS^c^	44.59	6.13
Seasonal ARIMA^d^	47.17	6.15

^a^RMSE: root mean squared error.

^b^MAPE: mean absolute percentage error.

^c^H-W ETS: Holt-Winters exponential smoothing.

^d^ARIMA: autoregressive integrated moving average.

**Table 4:** Predicted values of notified pulmonary tuberculosis cases in the elderly using the exponential smoothing model and their actual values in 2021.

Month	Point estimation	80% CI	95% CI	Notified number
January	527	463-592	428-626	515
February	456	386-525	349-562	453
March	624	550-698	510-737	580
April	621	543-699	501-741	625
May	652	569-734	526-778	634
June	593	507-679	461-725	690
July	674	585-764	537-812	663
August	664	570-757	521-807	744
September	661	565-758	513-810	682
October	570	470-671	417-724	585
November	548	445-652	390-706	644
December	552	445-658	389-715	590

## Discussion

### Principal Findings

With the rising geriatric population, the increasing incidence and mortality of PTB emerged as a vital public health concern. Previous literature indicated that the increased TB incidence in older adults might be driven by the rising latent TB infections and weakened immunity [29]. Nevertheless, a comparatively developed province in Eastern China also faced a huge challenge in PTB control and prevention among the elderly. This study provides epidemiological evidence for scientific intervention and assessment of the PTB trend for this target population.

From the available epidemiological features of PTB in the elderly, the notified incidence demonstrated a significant downward trend with a reduction of 30%, whereas the bacteriologically positive incidence rate remained steady at approximately 60/100,000. The former was attributable to the deployment of the national directly observed treatment strategy combined with the internet surveillance system implemented in China since 2005, which could lower PTB transmission [10]. Moreover, increased collaboration among the designated hospitals, local Center for Disease Control and Prevention, and community health centers could improve the early identification of PTB cases, avoid delays in diagnoses causing further disease transmission, and facilitate prompt and adequate overall treatment. These possibilities may reduce PTB morbidity in the general population and target groups like the elderly. Furthermore, since the introduction of the rapid molecular testing systems for PTB such as GeneXpert MTB/RIF, the turnaround time for determining PTB has been shortened; rifampicin resistance is promptly determined, and the sensitivity and specificity are higher compared to those of classic smear microscopy, which could identify the number of etiologically positive cases despite the gradual decrease in the total incidence in this specific group [30].

From the geographical distribution, Quzhou and Jinhua Cities showed a higher PTB incidence in the elderly. In recent years, several investigations on PTB incidence have been performed in these regions. Considering the lack of PTB knowledge or the probable inability to seek care in this target group, community-based active screening for PTB in Quzhou had proved its efficacy in locally reducing the epidemic [31]. Thus, for areas with special funds available, several rounds of active PTB screening should be considered for successive identification of active PTB cases through different methods such as symptom screening, chest radiography, or molecular rapid diagnostic tests, used alone or in combination [32]. Meanwhile, for other areas, screening could be implemented in combination with the existing basic public health service project in China, which includes annual checkup for symptoms and chest radiography in the elderly [33]. Thus, strengthening the assessment through physical checkups is necessary to refer and trace elderly individuals with chest radiography abnormalities in a timely manner.

From our findings, the sex ratio in the elderly was 2.66:1 (male:female), which was slightly higher than that in the general population in Zhejiang Province [12]. Although available evidence from various countries indicated a higher susceptibility of men to PTB, the differences in age might be attributed to sociocultural roles, behaviors, and changes in the immune function among the sexes [34,35]. More PTB cases in the elderly were clustered in the age group of 65-70 years. As these people are typically free, they can form more clusters of infection. Therefore, determining active PTB cases particularly in the early stages of the disease among the elderly may be crucial for interventions and reversal of this epidemic, and additional health policies should be considered, such as inpatient isolation treatment with full reimbursement through medical insurance to reduce transmission and improve adherence to PTB treatment.

One study in Germany has reported significantly lower drug resistance rates in the elderly than the younger TB group for all TB drugs (6.5% vs 13.9%) and multidrug resistance (0.6% vs 3.1%) in 2011 [36]. Our results revealed that the drug resistance rate in the elderly was 5.86% in Zhejiang Province. Considering the reactivation of previous latent infection, the drug resistance rate in the elderly might be lower than that in the general group [4,35].

A delay in PTB diagnoses was identified, which implies the lack of knowledge on PTB’s signs and symptoms in this specific group, thereby leading to a potential delay in seeking treatment. Moreover, acquiring sufficient diagnostic and treatment services for the geriatric population may be inconvenient. Therefore, succinct health education for PTB should be advocated in the elderly communities and increasing health promotions for this target group should be considered. Furthermore, a green channel in outpatient services should be provided to the elderly population to offer them a more convenient medical treatment experience.

Further, a prolonged treatment course (>9 months) was common in the elderly. Existing studies have revealed that older patients were more likely to experience drug-induced adverse reactions, such as hepatotoxicity and acute kidney injury during the treatment duration [37-39]. Furthermore, several complications and comorbidities including diabetes mellitus were prevalent in the aging group [40]. These factors might lead to the extension of standardized short-course chemotherapy.

Here, in predicting the PTB epidemic in the elderly based on the LRM, the goal of reducing PTB morbidity to 50% might be achieved by the middle of 2024. The ongoing efforts and implementations in Zhejiang Province may be effective in controlling PTB prevalence in the aging population. Due to limited data on PTB deaths and a significant decline in PTB-related deaths in 2020, we did not create a trend forecast. The significant decline might be attributed to the prevalence of the ongoing COVID-19 pandemic, which has influenced lifestyle habits such as wearing masks regularly. It would also be highly influential in preventing respiratory infectious diseases like PTB. Meanwhile, we used the optimized H-W ETS model to predict the PTB number in 2021 among the elderly, suggesting a comparatively better effect for identifying further trends. This result also implied that the H-W ETS model could provide a more elaborate atlas and consequently offer a basis for further policy developments for this specific group.

However, this study had some limitations. First, the PTB epidemiology between the general population and the elderly was not compared, which might influence other findings for these specific groups. Second, the predictive model did not include other details such as health policies, influence of the COVID-19 pandemic, and preventive methods.

### Conclusions

With the global aging population and inevitable challenge of active PTB cases in the elderly, more comprehensive and effective interventions such as active PTB screening combined with physical checkup and succinct health education should be implemented in this specific group. Meanwhile, a more systematic assessment of the PTB epidemic trend in the elderly should be considered to incorporate more predictive factors.

## Abbreviations

ARIMA: autoregressive integrated moving average
ETS: exponential smoothing
GCM: growth curve model
H-W ETS: Holt-Winters exponential smoothing
LRM: linear regression model
MAPE: mean absolute percentage error
PTB: pulmonary tuberculosis
RMSE: root mean squared error
TBIMS: TB information management system
WHO: World Health Organization
